# Scaling Relations and Self-Similarity of 3-Dimensional Reynolds-Averaged Navier-Stokes Equations

**DOI:** 10.1038/s41598-017-06669-z

**Published:** 2017-07-25

**Authors:** Ali Ercan, M. Levent Kavvas

**Affiliations:** 1 J. Amorocho Hydraulics Laboratory, Department of Civil and Environmental Engineering, University of California, Davis, CA 95616 USA; 2 Hydrologic Research Laboratory and J. Amorocho Hydraulics Laboratory, Department of Civil and Environmental Engineering, University of California, Davis, CA 95616 USA

## Abstract

Scaling conditions to achieve self-similar solutions of 3-Dimensional (3D) Reynolds-Averaged Navier-Stokes Equations, as an initial and boundary value problem, are obtained by utilizing Lie Group of Point Scaling Transformations. By means of an open-source Navier-Stokes solver and the derived self-similarity conditions, we demonstrated self-similarity within the time variation of flow dynamics for a rigid-lid cavity problem under both up-scaled and down-scaled domains. The strength of the proposed approach lies in its ability to consider the underlying flow dynamics through not only from the governing equations under consideration but also from the initial and boundary conditions, hence allowing to obtain perfect self-similarity in different time and space scales. The proposed methodology can be a valuable tool in obtaining self-similar flow dynamics under preferred level of detail, which can be represented by initial and boundary value problems under specific assumptions.

## Introduction

Dimensional analysis^1–6^, power law behavior^1, 5–8^, fractals^9, 10^, and multi-fractals^11–14^ are related notions that have been applied in various fields of science in general and fluid flow processes^15–17^ in particular to model the evolution of complex but self-similar dynamics under different spatial and temporal scales.

By means of dimensional analysis^1–6^ dimensionless products can be formed to reduce the number of variables to be considered. Various applications of dimensional analysis in engineering and physics can be found in Sedov^3^ and Barenblatt^5^. Originally intended to explain the power-law behavior of the low-frequency power spectra over a wide range of time scales^18^ and their connection with self-similar spatial structures (i.e. fractals^9^), self-organized criticality was introduced by Bak *et al*.^15, 16^ to explain the spatiotemporal scaling phenomena in nonequilibrium systems that display long-range correlations. The term fractal, the degree of irregularity or fragmentation which is identical at all geometric scales, was introduced by Mandelbrot^9, 10^. Long before the introduction of the fractals, mathematicians have known of scale invariant objects, such as Hilbert curve, or Koch curve, mainly due to their non-differentiability property^17^. Self-similarity property of such mathematical objects become popular especially after the pioneering works of Mandelbrot^9, 10^ on fractals.

An interesting feature of the fully developed turbulence is the possible existence of universal scaling behavior of small scale fluctuations (She and Leveque^19^; Benzi *et al*.^20^; and the references therein). Richardson^21^ explained the energy transfer from large to small scale eddies by the concept of self-similar cascades. Kolmogorov^7^ investigated energy distribution among eddies of the inertial range of isotropic flows and showed that the energy spectrum follows a power law scaling of order −5/3. Kolmogorov’s theory^7^ also predicts that the scaling between the velocity increments Δ*U*(*r*) = *U*(*x* + *r*) − *U*(*x*) at two points separated by a distance r (*η *≪ *r *≪ *L*) as 〈Δ*U*(*r*)^*n*^〉 ≈ *r* ^*ς*(*n*)^ with *ς*(*n*) = *n*/3 in the fully developed regime. Here, *η* is the dissipation scale and L is the integral scale, and 〈〉 is the average over the probability density of Δ*U*(*r*). Experimental and numerical studies^22, 23^ demonstrated that the *ς*(*n*) deviates from n/3 for n > 3 due to strong intermittent character of the energy dissipation. With the concept of extended self-similarity, Benzi *et al*.^20^ made noteworthy progress in accurately estimating the scaling exponents. Furthermore, they showed that the statistical properties of turbulence could be self-similar at also low Reynolds numbers by the same set of scaling exponents of the fully developed regime.

Here, self-similarity conditions of 3D Reynolds-Averaged Navier-Stokes Equations, as an initial and boundary value problem, are obtained based on the relations between scaling exponents of the flow variables by utilizing Lie Group of Point Scaling Transformations. In the nineteenth century, Sophus Lie developed the theory of continuous groups (or Lie groups) of transformations, which can be characterized by infinitesimal generators admitted by a given differential equation. Invariant or similarity solutions can be found if a partial differential equation is invariant under a Lie group. Among others, Bluman and Cole^24^, Schwarz^25^, Ibragimov^26, 27^, Bluman and Anco^28^, and Polyanin and Manzhirov^29^ provided algorithms to find infinitesimal generators for various applications of Lie groups.

In recent years, one-parameter Lie group of point scaling transformations were applied to investigate scale invariance and self-similarity conditions of various hydrologic and hydraulic problems. Haltas and Kavvas^30^ investigated the scale invariance conditions of a variety of one dimensional hydrologic problems including confined and unconfined aquifer groundwater flows. The self-similarity conditions of one-dimensional unsteady open channel flow^31^, one dimensional suspended sediment transport^32^, and two-dimensional depth averaged flow^33^ processes were investigated with numerical examples. More recently, Ercan and Kavvas^34^ derived the self-similarity conditions of 3-dimensional incompressible Navier-Stokes equations for Newtonian fluids but without numerical or experimental demonstration.

Within the above framework, the objectives of this article are (1) to derive the self-similarity conditions of 3-dimensional Reynolds Averaged Navier-Stokes equations closed by the standard k-*ε* turbulence model by applying one-parameter Lie group of point scaling transformations, and (2) to perform state of the art computational fluids dynamics simulations to demonstrate similitude or self-similarity of 3-dimensional flow dynamics under various spatial and temporal scales.

## Theory and Methods

The one-parameter Lie group of point scaling transformations can be defined by

1 $$\sigma ={\beta }^{{\alpha }_{\sigma }}\bar{\sigma }$$

which maps the variable σ in the original space to the variable $\bar{\sigma }$ in the scaled space. Here, β is the scaling parameter and α_σ_ is the scaling exponent of the variable σ. Scaling ratio of the parameter σ can be defined as ${\sigma }_{r}=\frac{\sigma }{\bar{\sigma }}={\beta }^{{\alpha }_{\sigma }}$. Reynolds Averaged Navier-Stokes equations^35^ for incompressible Newtonian flows can be written in Cartesian coordinate system as

2 $$\frac{\partial {U}_{i}}{\partial {x}_{i}}=0$$

3 $$\frac{\partial {U}_{i}}{\partial t}+{U}_{j}\frac{\partial {U}_{i}}{\partial {x}_{j}}={g}_{i}-\frac{1}{\rho }\frac{\partial p}{\partial {x}_{i}}+\frac{\partial }{\partial {x}_{j}}(\upsilon \frac{\partial {U}_{i}}{\partial {x}_{j}}-\langle {u}_{i}{u}_{j}\rangle )$$

where t is time, *x*_*i*_ is the i-coordinate in Cartesian coordinate system where i = 1, 2, 3, *U*_*i*_ is the averaged flow velocity in i-coordinate, *u*_*i*_ is the fluctuating velocity in i-coordinate, *p* is the averaged pressure, *υ* is the kinematic viscosity, *ρ* is the density of the fluid, *g*_*i*_ is the gravitational acceleration in i-coordinate. Different turbulence closures can be used to estimate Reynolds stresses 〈*u*_*i*_ *u*_*j*_〉. Based on the Boussinesq’s assumption of linear stress-strain relation, Reynolds stresses can be calculated as

4 $$-\langle {u}_{i}{u}_{j}\rangle ={\upsilon }_{t}(\frac{\partial {U}_{i}}{\partial {x}_{j}}+\frac{\partial {U}_{j}}{\partial {x}_{i}})-2/3k{\delta }_{ij}$$

For the case of standard k-*ε* turbulence closure, the eddy viscosity *υ*_*t*_ can be estimated as *υ*_*t*_ = *C*_*μ*_ *k* ^2^/*ε* where turbulent kinetic energy k and its rate of dissipation *ε* can be calculated from

5 $$\frac{\partial k}{\partial t}+{U}_{j}\frac{\partial k}{\partial {x}_{j}}={P}_{k}-\varepsilon +\frac{\partial }{\partial {x}_{j}}[(\upsilon +{\upsilon }_{t}/{\sigma }_{k})\frac{\partial k}{\partial {x}_{j}}]$$

6 $$\frac{\partial \varepsilon }{\partial t}+{U}_{j}\frac{\partial \varepsilon }{\partial {x}_{j}}={C}_{\varepsilon 1}\frac{\varepsilon }{k}{P}_{k}-{C}_{\varepsilon 2}\frac{{\varepsilon }^{2}}{k}+\frac{\partial }{\partial {x}_{j}}[(\upsilon +{\upsilon }_{t}/{\sigma }_{\varepsilon })\frac{\partial \varepsilon }{\partial {x}_{j}}]$$

where ${P}_{k}={u}_{t}\frac{\partial {U}_{i}}{\partial {x}_{j}}(\frac{\partial {U}_{i}}{\partial {x}_{j}}+\frac{\partial {U}_{j}}{\partial {x}_{i}})$ and the model coefficients^36^ are *C*_*μ*_ = 0.09, *C*_*ε*1_ = 1.44, *C*_*ε*2_ = 1.92, *σ*_*k*_ = 1.0, *σ*_*ε*_ = 1.3. Here, we selected standard k-*ε* turbulence closure since it is the most widely used turbulence model^37^.

Applying the one-parameter Lie scaling transformations, the Reynolds-Averaged Navier-Stokes equations for incompressible Newtonian flows (Equations 2 and 3) yield the below equations in the scaled domain

7 $${\beta }^{{\alpha }_{{U}_{i}}-{\alpha }_{{x}_{i}}}\frac{\partial {\bar{U}}_{i}}{\partial {\bar{x}}_{i}}=0\ldots $$

8 $$\begin{array}{c}{\beta }^{{\alpha }_{{U}_{i}}-{\alpha }_{t}}\frac{\partial {\bar{U}}_{i}}{\partial \bar{t}}+{\beta }^{{\alpha }_{{U}_{j}}+{\alpha }_{{U}_{i}}-{\alpha }_{{x}_{j}}}{\bar{U}}_{j}\frac{\partial {\bar{U}}_{i}}{\partial {\bar{x}}_{j}}={\beta }^{{\alpha }_{{g}_{i}}}{\bar{g}}_{i}-{\beta }^{{\alpha }_{p}-{\alpha }_{\rho }-{\alpha }_{{x}_{i}}}\frac{1}{\bar{\rho }}\frac{\partial \bar{p}}{\partial {\bar{x}}_{i}}\\ +{\beta }^{{\alpha }_{{U}_{i}}-2{\alpha }_{xj}+{\alpha }_{\upsilon }}\frac{\partial }{\partial {\bar{x}}_{j}}(\bar{\upsilon }\frac{\partial {\bar{U}}_{i}}{\partial {\bar{x}}_{j}})-{\beta }^{{\alpha }_{\langle {u}_{i}{u}_{j}\rangle }-{\alpha }_{{x}_{j}}}\frac{\partial }{\partial {\bar{x}}_{j}}\overline{\langle {u}_{i}{u}_{j}\rangle }\end{array}$$

Similarly, Reynolds stresses, turbulent kinetic energy, and its rate of dissipation in the transformed domain can be calculated from

9 $$-{\beta }^{{\alpha }_{\langle {u}_{i}{u}_{j}\rangle }}\overline{\langle {u}_{i}{u}_{j}\rangle }={\beta }^{{\alpha }_{{\upsilon }_{t}}}{\bar{\upsilon }}_{t}[{\beta }^{{\alpha }_{{U}_{i}}-{\alpha }_{{x}_{j}}}\frac{\partial {\bar{U}}_{i}}{\partial {\tilde{x}}_{j}}+{\beta }^{{\alpha }_{{U}_{j}}-{\alpha }_{{x}_{i}}}\frac{\partial {\bar{U}}_{j}}{\partial {\bar{x}}_{i}}]-2/3{\beta }^{{\alpha }_{k}}\bar{k}{\delta }_{ij}$$

10 $$\begin{array}{c}{\beta }^{{\alpha }_{k}-{\alpha }_{t}}\frac{\partial \bar{k}}{\partial \bar{t}}+{\beta }^{{\alpha }_{{U}_{j}}+{\alpha }_{k}-{\alpha }_{{x}_{j}}}{\bar{U}}_{j}\frac{\partial \bar{k}}{\partial {\bar{x}}_{j}}={\beta }^{{\alpha }_{{P}_{k}}}{\bar{P}}_{k}-{\beta }^{{\alpha }_{\varepsilon }}\bar{\varepsilon }+{\beta }^{{\alpha }_{\upsilon }+{\alpha }_{k}-2{\alpha }_{{x}_{j}}}\frac{\partial }{\partial {\bar{x}}_{j}}(\bar{\upsilon }\frac{\partial \bar{k}}{\partial {\bar{x}}_{j}})\\ +{\beta }^{{\alpha }_{{\upsilon }_{t}}+{\alpha }_{k}-2{\alpha }_{{x}_{j}}}\frac{\partial }{\partial {\bar{x}}_{j}}({\bar{\upsilon }}_{t}/{\sigma }_{k}\frac{\partial \bar{k}}{\partial {\bar{x}}_{j}})\end{array}$$

11 $$\begin{array}{c}{\beta }^{{\alpha }_{\varepsilon }-{\alpha }_{t}}\frac{\partial \bar{\varepsilon }}{\partial \bar{t}}+{\beta }^{{\alpha }_{{U}_{j}}+{\alpha }_{\varepsilon }-{\alpha }_{{x}_{j}}}{\bar{U}}_{j}\frac{\partial \bar{\varepsilon }}{\partial {\bar{x}}_{j}}={\beta }^{{\alpha }_{\varepsilon }-{\alpha }_{k}+{\alpha }_{{P}_{k}}}{C}_{\varepsilon 1}\frac{\bar{\varepsilon }}{\bar{k}}{\bar{P}}_{k}-{\beta }^{2{\alpha }_{\varepsilon }-{\alpha }_{k}}{C}_{\varepsilon 2}\frac{{\bar{\varepsilon }}^{2}}{\bar{k}}\\ +{\beta }^{{\alpha }_{\upsilon }+{\alpha }_{\varepsilon }-2{\alpha }_{{x}_{j}}}\frac{\partial }{\partial {\bar{x}}_{j}}(\bar{\upsilon }\frac{\partial \bar{\varepsilon }}{\partial {\bar{x}}_{j}})+{\beta }^{{\alpha }_{{\upsilon }_{t}}+{\alpha }_{\varepsilon }-2{\alpha }_{{x}_{j}}}\frac{\partial }{\partial {\bar{x}}_{j}}({\bar{\upsilon }}_{t}/{\sigma }_{\varepsilon }\frac{\partial \bar{\varepsilon }}{\partial {\bar{x}}_{j}})\end{array}$$

where $${\beta }^{{\alpha }_{{\upsilon }_{t}}}{\bar{\upsilon }}_{t}={C}_{\mu }{\beta }^{{\alpha }_{2k}-{\alpha }_{\varepsilon }}{\bar{k}}^{2}/\bar{\varepsilon }$$.

The self-similarity conditions for the Reynolds-Averaged Navier-Stokes equations for incompressible flows can be found when the IBVP of the flow process in the prototype domain, subjected to the Lie group of point scaling transformations, remains invariant in the transformed variables, as listed below:

12 $${\alpha }_{{U}_{1}}-{\alpha }_{{x}_{1}}={\alpha }_{{U}_{2}}-{\alpha }_{{x}_{2}}={\alpha }_{{U}_{3}}-{\alpha }_{{x}_{3}}$$

13 $$\begin{array}{rcl}{\alpha }_{{u}_{1}}-{\alpha }_{t} & = & {\alpha }_{{u}_{1}}+{\alpha }_{{u}_{1}}-{\alpha }_{{x}_{1}}={\alpha }_{{u}_{2}}+{\alpha }_{{u}_{1}}-{\alpha }_{{x}_{2}}={\alpha }_{{u}_{3}}+{\alpha }_{{u}_{1}}-{\alpha }_{{x}_{3}}={\alpha }_{p}-{\alpha }_{\rho }-{\alpha }_{{x}_{1}}\\  & = & {\alpha }_{{g}_{1}}={\alpha }_{{U}_{1}}-2{\alpha }_{{x}_{1}}+{\alpha }_{\upsilon }={\alpha }_{{U}_{1}}-2{\alpha }_{{x}_{2}}+{\alpha }_{\upsilon }={\alpha }_{{U}_{1}}-2{\alpha }_{{x}_{3}}+{\alpha }_{\upsilon }\\  & = & {\alpha }_{\langle {u}_{1}{u}_{1}\rangle }-{\alpha }_{{x}_{1}}={\alpha }_{\langle {u}_{1}{u}_{2}\rangle }-{\alpha }_{{x}_{2}}={\alpha }_{\langle {u}_{1}{u}_{3}\rangle }-{\alpha }_{{x}_{3}}\end{array}$$

14 $$\begin{array}{rcl}{\alpha }_{{u}_{2}}-{\alpha }_{t} & = & {\alpha }_{{u}_{1}}+{\alpha }_{{u}_{2}}-{\alpha }_{{x}_{1}}={\alpha }_{{u}_{2}}+{\alpha }_{{u}_{2}}-{\alpha }_{{x}_{2}}={\alpha }_{{u}_{3}}+{\alpha }_{{u}_{2}}-{\alpha }_{{x}_{3}}={\alpha }_{p}-{\alpha }_{\rho }-{\alpha }_{{x}_{2}}\\  & = & {\alpha }_{{g}_{2}}={\alpha }_{{U}_{2}}-2{\alpha }_{{x}_{1}}+{\alpha }_{\upsilon }={\alpha }_{{U}_{2}}-2{\alpha }_{{x}_{2}}+{\alpha }_{\upsilon }={\alpha }_{{U}_{2}}-2{\alpha }_{{x}_{3}}+{\alpha }_{\upsilon }\\  & = & {\alpha }_{\langle {u}_{2}{u}_{1}\rangle }-{\alpha }_{{x}_{1}}={\alpha }_{\langle {u}_{2}{u}_{2}\rangle }-{\alpha }_{{x}_{2}}={\alpha }_{\langle {u}_{2}{u}_{3}\rangle }-{\alpha }_{{x}_{3}}\end{array}$$

15 $$\begin{array}{rcl}{\alpha }_{{u}_{3}}-{\alpha }_{t} & = & {\alpha }_{{u}_{1}}+{\alpha }_{{u}_{3}}-{\alpha }_{{x}_{1}}={\alpha }_{{u}_{2}}+{\alpha }_{{u}_{3}}-{\alpha }_{{x}_{2}}\\  & = & {\alpha }_{{u}_{3}}+{\alpha }_{{u}_{3}}-{\alpha }_{{x}_{3}}={\alpha }_{p}-{\alpha }_{\rho }-{\alpha }_{{x}_{3}}\\  & = & {\alpha }_{{g}_{3}}={\alpha }_{{U}_{3}}-2{\alpha }_{{x}_{1}}+{\alpha }_{\upsilon }\\  & = & {\alpha }_{{U}_{3}}-2{\alpha }_{{x}_{2}}+{\alpha }_{\upsilon }={\alpha }_{{U}_{3}}-2{\alpha }_{{x}_{3}}+{\alpha }_{\upsilon }\\  & = & {\alpha }_{\langle {u}_{3}{u}_{1}\rangle }-{\alpha }_{{x}_{1}}={\alpha }_{\langle {u}_{3}{u}_{2}\rangle }-{\alpha }_{{x}_{2}}={\alpha }_{\langle {u}_{3}{u}_{3}\rangle }-{\alpha }_{{x}_{3}}\end{array}$$

From the equalities in Equations 13–15, the scaling exponents of the length dimensions in i = 1,2,3 coordinates can be deduced as

16 $${\alpha }_{{x}_{1}}={\alpha }_{{x}_{2}}={\alpha }_{{x}_{3}}={\alpha }_{x}=\frac{{\alpha }_{\upsilon }+{\alpha }_{t}}{2}$$

In other words, the scaling exponents of length dimensions in i = 1,2,3 coordinates must be equal (${\alpha }_{{x}_{1}}={\alpha }_{{x}_{2}}={\alpha }_{{x}_{3}}={\alpha }_{x}$) since the viscosity is constant in i = 1,2,3 coordinates. Similarly, the scaling exponents of velocity in i = 1,2,3 coordinates can be obtained as

17 $${\alpha }_{{u}_{1}}={\alpha }_{{u}_{2}}={\alpha }_{{u}_{3}}={\alpha }_{u}={\alpha }_{x}-{\alpha }_{t}=\frac{{\alpha }_{\upsilon }-{\alpha }_{t}}{2}$$

Furthermore, the scaling exponents of gravity in i = 1,2,3 coordinates and pressure can be written in terms of the scaling exponents of length, time, and density as

18 $${\alpha }_{{g}_{1}}={\alpha }_{{g}_{2}}={\alpha }_{{g}_{3}}={\alpha }_{g}={\alpha }_{x}-2{\alpha }_{t}\ldots $$

19 $${a}_{p}=2{\alpha }_{x}-2{\alpha }_{t}+{\alpha }_{\rho }\ldots $$

For Equations (10–11) to be invariant, below equalities must hold

20 $$\begin{array}{c}{\alpha }_{k}-{\alpha }_{t}={\alpha }_{{U}_{1}}+{\alpha }_{k}-{\alpha }_{{x}_{1}}={\alpha }_{{U}_{2}}+{\alpha }_{k}-{\alpha }_{{x}_{2}}={\alpha }_{{U}_{3}}+{\alpha }_{k}-{\alpha }_{{x}_{3}}={\alpha }_{{P}_{k}}={\alpha }_{\varepsilon }\\ \quad \quad \quad \,\,\,=\,{\alpha }_{\upsilon }+{\alpha }_{k}-2{\alpha }_{{x}_{1}}={\alpha }_{\upsilon }+{\alpha }_{k}-2{\alpha }_{{x}_{2}}={\alpha }_{\upsilon }+{\alpha }_{k}-2{\alpha }_{{x}_{3}}\end{array}$$

21 $$\begin{array}{c}{\alpha }_{\varepsilon }-{\alpha }_{t}={\alpha }_{{U}_{1}}+{\alpha }_{\varepsilon }-{\alpha }_{{x}_{1}}={\alpha }_{{U}_{2}}+{\alpha }_{\varepsilon }-{\alpha }_{{x}_{2}}={\alpha }_{{U}_{3}}+{\alpha }_{\varepsilon }-{\alpha }_{{x}_{3}}={\alpha }_{\varepsilon }-{\alpha }_{k}+{\alpha }_{{P}_{k}}\\ \quad \quad \quad \,\,\,=\,2{\alpha }_{\varepsilon }-{\alpha }_{k}={\alpha }_{\upsilon }+{\alpha }_{\varepsilon }-2{\alpha }_{{x}_{1}}={\alpha }_{\upsilon }+{\alpha }_{\varepsilon }-2{\alpha }_{{x}_{2}}={\alpha }_{\upsilon }+{\alpha }_{\varepsilon }-2{\alpha }_{{x}_{3}}\end{array}$$

22 $${\alpha }_{{\upsilon }_{t}}={\alpha }_{2k}-{\alpha }_{\varepsilon }={\alpha }_{\upsilon }\ldots $$

For Equation (9) to be invariant, scaling exponents of Reynolds stresses can be obtained as

23 $${\alpha }_{\langle {u}_{i}{u}_{j}\rangle }=2{\alpha }_{x}-2{\alpha }_{t}={\alpha }_{k}\ldots $$

As a result, the scaling exponents that are obtained by the one-parameter Lie group of point scaling transformations for the variables of the 3D Reynolds Averaged Navier-Stokes equations and the variables of its k - ε turbulence closure are tabulated in Tables 1 and 2, respectively. The initial and boundary conditions of the 3D Reynolds Averaged Navier-Stokes equations can be transformed with respect to Lie group of point scaling similar to Chapter IV in Ercan and Kavvas^34^.

**Table 1:** The scaling exponents obtained by the one-parameter Lie group of point scaling transformations for the variables of the 3D Reynolds Averaged Navier-Stokes equations.

Variable	Scaling Conditions in terms of *α*_*x*_, *α*_*t*_ and *α*_*ρ*_	Scaling Conditions in terms of *α*_*x*_ and *α*_*ρ*_; under the same gravity
Length x_i_ (in i = 1, 2, 3 coordinates)	${\alpha }_{{x}_{i}}={\alpha }_{x}$	*α*_*x*_
Time, t	*α*_*t*_	*α*_*t*_ = *α*_*x*_/2
Density of fluid, *ρ*	*α*_*ρ*_	*α*_*ρ*_
Average Flow velocity U_i_	${\alpha }_{{U}_{i}}={\alpha }_{U}={\alpha }_{x}-{\alpha }_{t}$	*α*_*U*_ = *α*_*x*_/2
Pressure, p	*α*_*p*_ = *α*_*ρ*_ + 2*α*_*x*_ − 2*α*_*t*_	*α*_*p*_ = *α*_*x*_ + *α*_*ρ*_
Kinematic viscosity, *υ*	*α*_*υ*_ = 2*α*_*x*_ − *α*_*t*_	*α*_*υ*_ = 3*α*_*x*_/2
Gravitational acceleration, g_i_	*α*_*g*_ = *α*_*x*_ − 2*α*_*t*_	*α*_*g*_ = 0
Reynolds stresses, 〈*u*_*i*_ *u*_*j*_〉	${\alpha }_{\langle {u}_{i}{u}_{j}\rangle }=2{\alpha }_{x}-2{\alpha }_{t}$	${\alpha }_{\langle {u}_{i}{u}_{j}\rangle }={\alpha }_{x}$

**Table 2:** The scaling conditions obtained by the one-parameter Lie group of point scaling transformations for the variables of the three-dimensional k - ε turbulence model.

Variable	Scaling Conditions in terms of α_x_ and α_t_	Scaling Conditions in terms of α_*x*_ and α_*ρ*_; under the same gravity
Eddy viscosity, *υ*_*t*_	${{\rm{\alpha }}}_{{{\rm{\upsilon }}}_{{\rm{t}}}}=2{{\rm{\alpha }}}_{{\rm{x}}}-{{\rm{\alpha }}}_{{\rm{t}}}$	${{\rm{\alpha }}}_{{{\rm{\upsilon }}}_{{\rm{t}}}}=3{{\rm{\alpha }}}_{{\rm{x}}}/2$
Turbulent kinetic energy, k	α_k_ = 2α_x_ − 2α_t_	α_k_ = α_x_
Dissipation, ε	α_ε_ = 2α_x_ − 3α_t_	α_ε_ = α_x_/2
Production of turbulence due to horizontal velocity gradients, P_k_	${{\rm{\alpha }}}_{{{\rm{P}}}_{{\rm{k}}}}=2{{\rm{\alpha }}}_{{\rm{x}}}-3{{\rm{\alpha }}}_{{\rm{t}}}$	α_P_ = α_x_/2

## Results

Now, let us explore the obtained self-similarity conditions numerically for the 3D lid-driven cavity flow, which is a typical benchmark problem for solvers of the Navier-Stokes equations^38–40^. Numerical simulations here are performed by OpenFoam Version 2.4.0 by solving Reynolds Averaged Navier Stokes equations closed by the k-epsilon turbulence model. First, the lid-driven cavity flow is simulated over a cubic domain with 0.1 m edge length, for a duration of 20 seconds, when the lid velocity is 1 m/s, and fluid viscosity is 0.00001 m^2^/s for the original domain (i.e. Domain 1, or D1). Stagnant initial velocities are assumed and the velocities close to solid walls are estimated by wall functions^37^ assuming smooth conditions.

**Figure 1:**
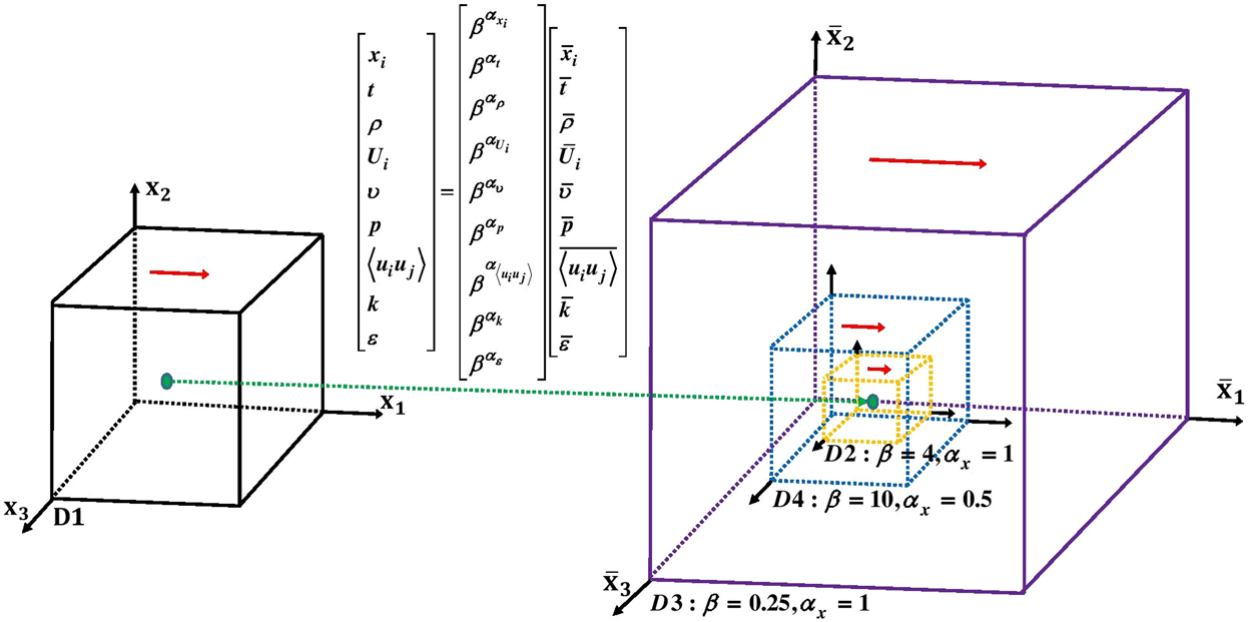
Schematic descriptions of the simulated self-similar cubic cavity flows (not to scale): original domain (D1) and its self-similar domains (D2, D3, and D4). Flow variables at specified time and space can be mapped to those at the corresponding time and space in the self-similar domains by means of Lie Group of scaling transformations. It is possible to obtain both larger and smaller domains by the selection of the scaling parameter β and the scaling exponent of length α_x_.

Utilizing the scaling exponents and ratios given in Table 3, which follow the scaling conditions provided in Tables 1 and 2, flow characteristics (cube edge lengths, simulation times, fluid viscosities, and lid velocities) of three self-similar domains (D2, D3, and D4) are obtained as presented in Table 4. Schematic descriptions of the original domain (D1) and the three self-similar domains (D2, D3, and D3) to simulate cubic cavity flow are demonstrated in Fig. 1. It is possible to obtain both larger (e.g. D3) and smaller (e.g. D2, and D4) self-similar domains by selecting the scaling parameter β and scaling exponent of length α_x_, which result in shorter (e.g. D3) and longer (e.g. D2, and D4) simulation times. A self-similar domain which is larger than the original domain (e.g. D3) can be obtained by selecting the scaling parameter β to be less than 1 and scaling exponent of length to be positive (which is equivalent to the case when the scaling parameter β is greater than 1 but scaling exponent of length α_x_ to be negative. For example, β = 0.25 and α_x_ = 1 is equivalent to β = 4 and α_x_ = −1 since both cases result in the same scaling ratio of 0.25).

**Table 3:** Scaling exponents and ratios to obtain Domains 2, 3, and 4 from Domain 1.

	Domain 2	Domain 3	Domain 4
Scaling parameter, *β*	4	0.25	10
scaling exponents			
Length, *α*_*x*_	1	1	0.5
Time, *α*_*t*_	0.5	0.5	0.25
Velocity, *α*_*U*_	0.5	0.5	0.25
Viscosity, *α*_*υ*_	1.5	1.5	0.75
Turbulent kinetic energy, *α*_*k*_	1	1	0.5
Dissipation, *α*_*ε*_	0.5	0.5	0.25
scaling ratios			
Length, ${\beta }^{{\alpha }_{x}}$	4	0.25	3.162
Time, ${\beta }^{{\alpha }_{t}}$	2	0.5	1.778
Velocity, ${\beta }^{{\alpha }_{U}}$	2	0.5	1.778
Viscosity, ${\beta }^{{\alpha }_{\upsilon }}$	8	0.125	5.623
Turbulent kinetic energy, ${\beta }^{{\alpha }_{k}}$	4	0.25	3.162
Dissipation, ${\beta }^{{\alpha }_{\varepsilon }}$	2	0.5	1.778

**Table 4:** Summary of the simulation characteristics for the original domain (Domain 1) and its self-similar domains (Domains 2, 3, and 4).

	Domain 1	Domain 2	Domain 3	Domain 4
Edge length of the cube (m)	0.1	0.025	0.4	0.0316
Simulation time (s)	20	10	40	11.2468
Lid velocity (m/s)	1	0.5	2	0.5623
Fluid viscosity (m^2^/s)	0.00001	0.00000125	0.00008	1.7783E-06

**Figure 2:**
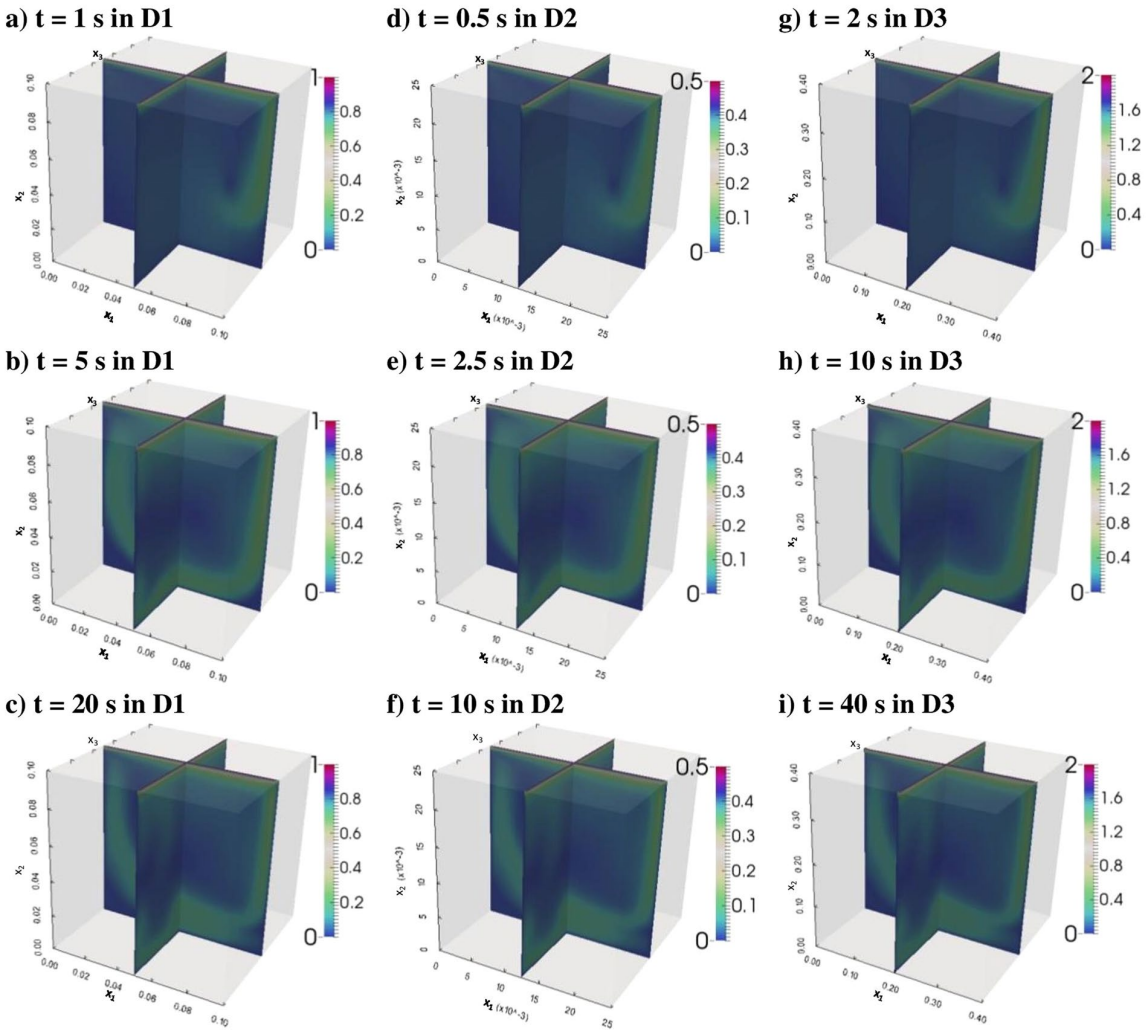
Contours of velocity magnitudes: at x = 0.05 m and z = 0.05 m in Domain 1 at simulation times of (**a**) 1 s, (**b**) 5 s, (**c**) 20 s; at x = 0.0125 m and z = 0.0125 m in Domain 2 at simulation times of (**d**) 0.5 s, (**e**) 2.5 s, (**f**) 10 s; at x = 0.2 m and z = 0.2 m in Domain 3 at simulation times of (**g**) 2 s, (**h**) 10 s, (**i**) 40 s. Velocity magnitudes within each row are self-similar to each other (Although the figures in each row look exactly similar, the edge lengths of the cubes and the velocity scales of the color bars are different for each domain, or column).

Contours of velocity magnitudes, $\sqrt{{U}_{1}^{2}+{U}_{2}^{2}+{U}_{3}^{2}}$, at cross-sections x = 0.05 m and z = 0.05 m in Domain 1 at simulation times of 1, 5, and 20 seconds are presented in the first column of Fig. 2. Similarly, velocity magnitudes at the corresponding two cross-sections in Domain 2 (at simulation times of 0.5 s, 2.5 s, 10 s), and those of Domain 3 (at simulation times of 2 s, 10 s, 40 s) are presented in the second and third columns of Fig. 2, respectively. Then, the velocity contours within each row of Fig. 2 are self-similar to each other for the corresponding simulation times in D1, D2, and D3. For example, as demonstrated in the first row of Fig. 2, the velocity contours of D1 at simulation time of 1 second are self-similar to those of D2 at simulation time of 0.5 second, and those of D3 at simulation time of 2 second.

**Figure 3:**
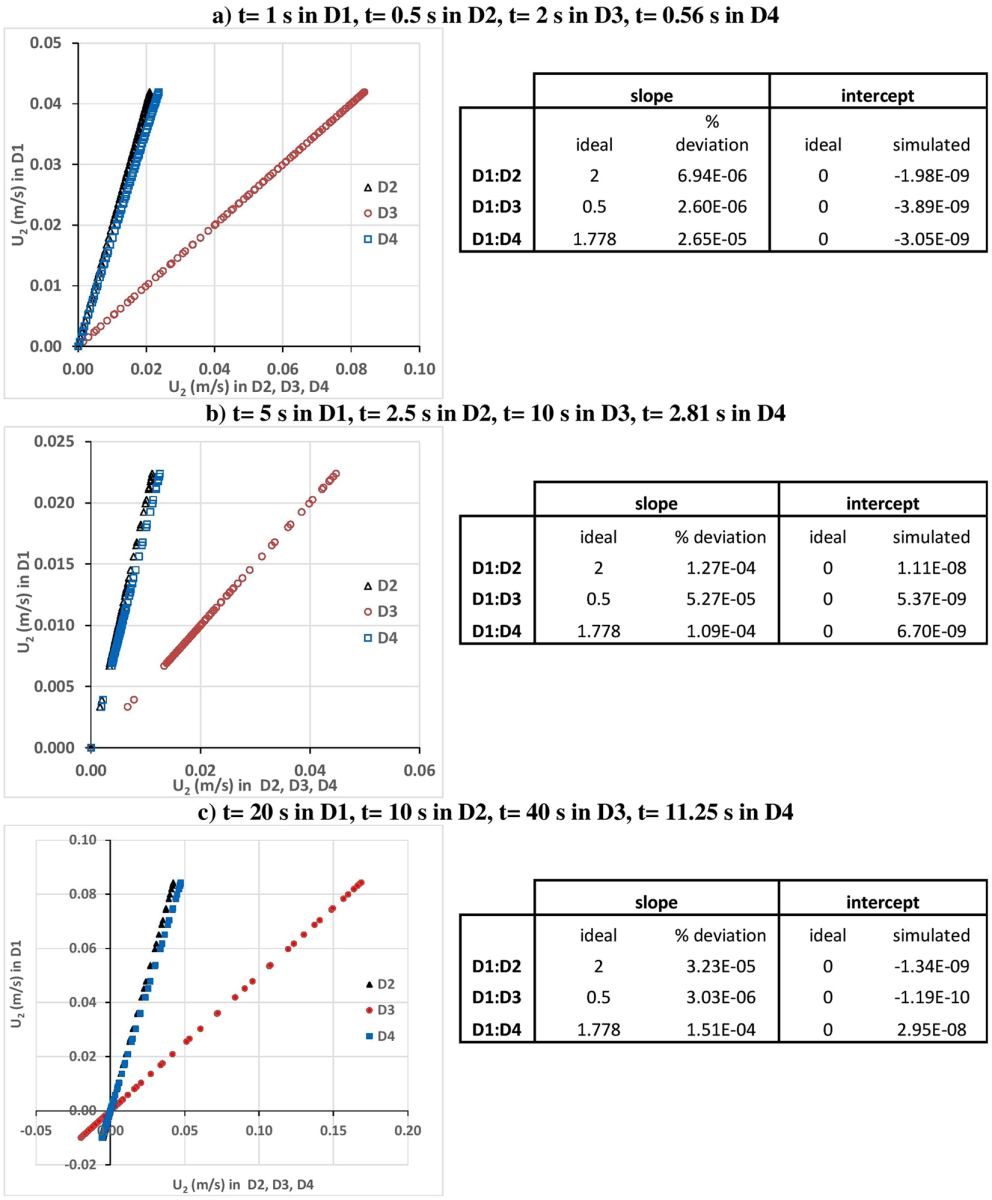
U_2_ at simulation times (**a**) 1, (**b**) 5 and (**c**) 20 seconds along the intersection line of planes x = 0.05 m and z = 0.05 m planes (i.e., the centerline of the cube in x_2_ direction) in domain 1 (D1) versus the corresponding velocities in domains 2, 3, and 4 (D2, D3, and D4). Simulation time of 1 s in D1 corresponds to 0.5 s in D2, 2 s in D3, and 0.56 s in D4. Simulation time of 5 s in D1 corresponds to 2.5 s in D2, 10 s in D3, and 2.81 s in D4. Simulation time of 20 s in D1 corresponds to 10 s in D2, 40 s in D3, and 11.25 s in D4.

**Figure 4:**
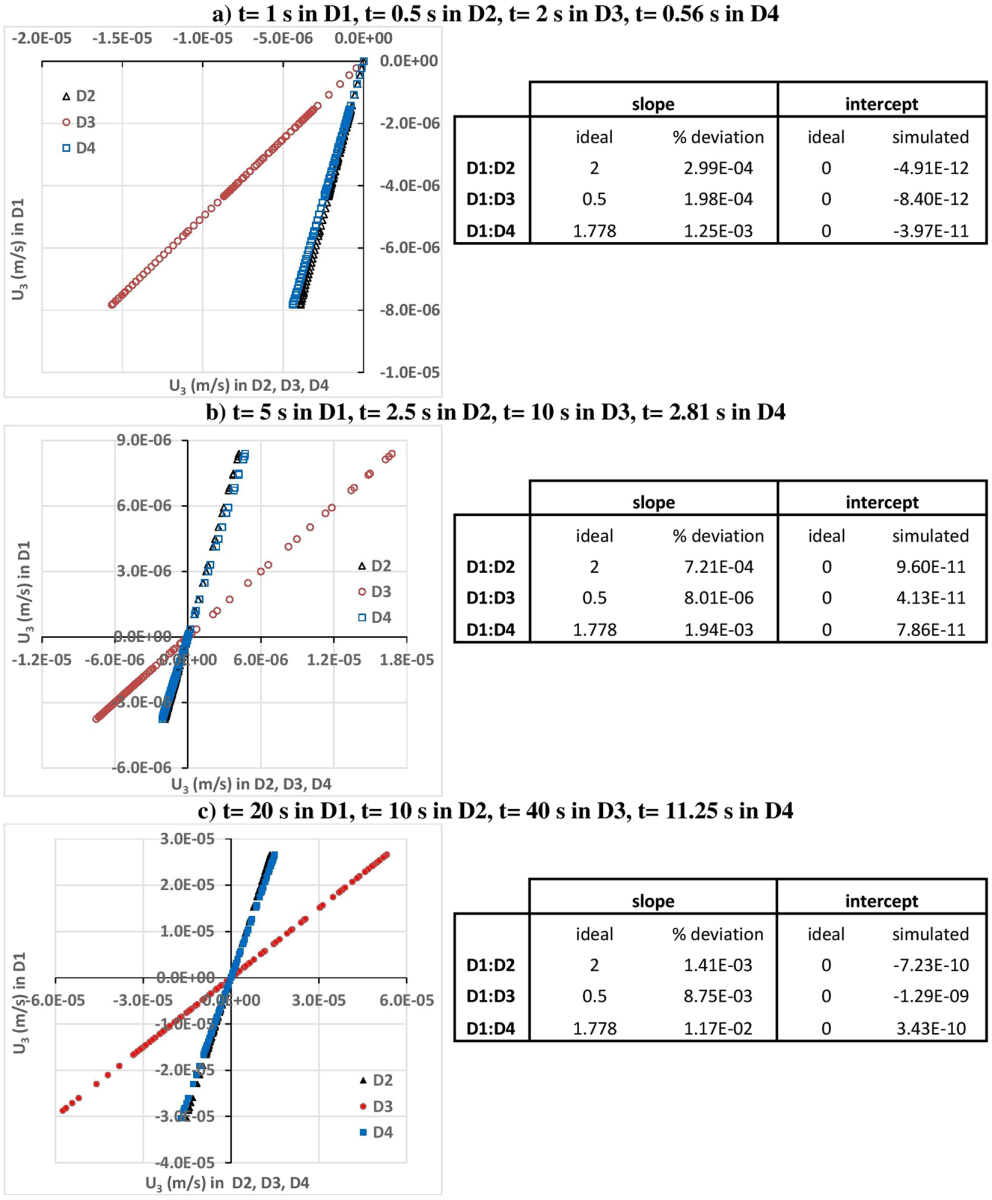
U_3_ at simulation times (**a**) 1, (**b**) 5 and (**c**) 20 seconds along the intersection line of planes x = 0.05 m and z = 0.05 m (i.e., the centerline of the cube in x_2_ direction) in domain 1 (D1) versus the corresponding velocities in domains 2, 3, and 4 (D2, D3, and D4). Simulation time of 1 s in D1 corresponds to 0.5 s in D2, 2 s in D3, and 0.56 s in D4. Simulation time of 5 s in D1 corresponds to 2.5 s in D2, 10 s in D3, and 2.81 s in D4. Simulation time of 20 s in D1 corresponds to 10 s in D2, 40 s in D3, and 11.25 s in D4.

Secondary velocities (velocities in i = 2, 3 directions, U_2_ and U_3_) at simulation times 1, 5 and 20 seconds along the intersection line of planes x = 0.05 m and z = 0.05 m (i.e., the centerline of the cube in x_2_ direction) in domain 1 (D1) versus the corresponding velocities in Domains 2–4 (D2, D3, and D4) are depicted in Figs 3 and 4 for different simulation times. Simulation time of 1 s in D1 corresponds to 0.5 s in D2, 2 s in D3, and 0.56 s in D4 (see Fig. 3a for U_2_, and Fig. 4a for U_3_). 5 s in D1 corresponds to 2.5 s in D2, 10 s in D3, and 2.81 s in D4 (see Fig. 3b for U_2_, and Fig. 4b for U_3_); and 20 s in D1 corresponds to 10 s in D2, 40 s in D3, and 11.25 s in D4 (see Fig. 3c for U_2_, and Fig. 4c for U_3_). In the case of perfect self-similarity, the plotted velocities in Figs 3 and 4 should follow perfect lines with slopes being the velocity scaling ratios ${\beta }^{{\alpha }_{U}}$(2 for D1/D2, 0.5 for D1/D3, and 1.778279 for D1/D4), and with intercept being 0. In order to check if the perfect self-similarity is reached or not, the slopes and the intercepts of the linear fits are estimated and percent deviations between simulated slopes and the ideal slopes (|ideal-simulated|/ideal × 100) and between ideal and simulated intercepts are tabulated next to each figure. As presented in Figs 3 and 4, percent deviations between simulated slopes and the ideal slopes vary between 1.51E-04 and 2.60E-06 for U_2_ and between 1.17E-02 and 8.01E-06 for U_3_. Simulated intercepts vary between −3.89E-09 and 2.95E-08 for U_2_ and between −1.29E-09 and 3.43E-10 for U_3_. These error estimates confirm near-perfect self-similarity between the secondary velocities of D1, D2, D3, and D4, through time.

Furthermore, Nash-Sutcliffe efficiency values are also estimated with respect to five flow variables at the end of the simulation: turbulent kinetic energy k, dissipation ε, and velocity components in 1, 2, 3 directions (U_1_, U_2_, and U_3_). Nash-Sutcliffe efficiency values are calculated between the variables of the original domain (k, ε, U_1_, U_2_, and U_3_), and their corresponding transformed variables (${{\rm{\beta }}}^{{{\rm{\alpha }}}_{{\rm{k}}}}\bar{{\rm{k}}}$, ${{\rm{\beta }}}^{{{\rm{\alpha }}}_{{\rm{\varepsilon }}}}\bar{{\rm{\varepsilon }}}$, ${{\rm{\beta }}}^{{{\rm{\alpha }}}_{{\rm{U}}}}{\bar{{\rm{U}}}}_{1},$ ${{\rm{\beta }}}^{{{\rm{\alpha }}}_{{\rm{U}}}}{\bar{{\rm{U}}}}_{2},$ ${{\rm{\beta }}}^{{{\rm{\alpha }}}_{{\rm{U}}}}{\bar{{\rm{U}}}}_{3}$) for Domains 2–4 at the 64000 (40 × 40 × 40) computational nodes. As tabulated in Table 5, the Nash-Sutcliffe efficiency values are between 0.999999999906 and 1 (the ideal value is 1), which confirm near perfect self-similarity between the flows of D1, D2, D3, and D4.

**Table 5:** Nash-Sutcliffe efficiency values between the flow variables of the original domain (D1) at t = 20 s and those of Domain 2 (D2) at t = 10 s, Domain 3 (D3) at t = 40 s, and Domain 4 (D4) at 11.25 s.

Domain	Dissipation, ε	Turbulent kinetic energy, k	U_1_	U_2_	U_3_
D2	0.999999999998	0.999999999995	0.999999999996	0.999999999995	0.999999999996
D3	0.999999999999	1.000000000000	0.999999999999	0.999999999999	0.999999999999
D4	0.999999999995	0.999999999950	0.999999999984	0.999999999981	0.999999999906

Nash-Sutcliffe efficiency values are calculated between the variables of the original domain (k, ε, U_1_, U_2_, and U_3_), and their corresponding transformed variables (${{\rm{\beta }}}^{{{\rm{\alpha }}}_{{\rm{k}}}}\bar{{\rm{k}}}$, ${{\rm{\beta }}}^{{{\rm{\alpha }}}_{{\rm{\varepsilon }}}}\bar{{\rm{\varepsilon }}}$, ${{\rm{\beta }}}^{{{\rm{\alpha }}}_{{\rm{U}}}}{\bar{{\rm{U}}}}_{1},$ ${{\rm{\beta }}}^{{{\rm{\alpha }}}_{{\rm{U}}}}{\bar{{\rm{U}}}}_{2},$ ${{\rm{\beta }}}^{{{\rm{\alpha }}}_{{\rm{U}}}}{\bar{{\rm{U}}}}_{3}$) for Domains 2–4 at the 64000 (40 × 40 × 40) computational nodes.

The Reynolds (Re) number is 10,000 (based on the lid velocity and the edge length of the cube) for the numerical simulations of D1, D2, D3, and D4. Four additional simulations, for Re = 20,000, are also performed when the lid velocities are twice of those in D1, D2, D3, and D4 (the other flow characteristics in Table 4 are kept the same). Near-perfect self-similarity for Re = 20,000 are again obtained. Nash-Sutcliffe efficiency values for turbulent kinetic energy k, dissipation ε, and velocity components in 1,2,3 directions at the end of the simulations are greater than 0.999999999894, showing near-perfect self-similarity. This finding was expected because the scaling conditions provided in Tables 1 and 2 do not depend on the Reynolds number of the flow.

## Discussion and Concluding Remarks

The sources of limitations in the numerical results include but are not limited to the Reynolds averaging process of the Navier Stokes equations, assumptions in the usage of the k-epsilon turbulence closure, the treatment of the near wall velocities by the wall functions under the assumption of smooth surfaces, the discretization of the numerical domain by uniform 40 × 40 × 40 cells, etc. Although the k-epsilon turbulence closure considered in this study is the most widely used model and showed its success especially in industrial engineering applications, it does not perform quite well in some unconfined flows, flows with large extra strains (e.g. curved boundary layers, swirling flows), rotating flows, and flows driven by anisotropy of normal Reynolds stresses^37^. Although the numerical simulations here inherit the limitations of the considered 3D Reynolds averaged Navier Stokes equations closed by k-epsilon turbulence model, we demonstrated that near-perfect self-similar solutions are achievable if the scaling conditions based on the Lie group similarity transformations are followed for specified governing equations and initial and boundary conditions.

As 3D Reynolds Averaged Navier-Stokes equations are time averaged forms of general Navier-Stokes equations (self-similarity of which were investigated in Ercan and Kavvas^34^), it is not surprising that the self-similarity conditions for both equation systems are consistent with respect to the main flow variables (x_i_, t, ρ, U_i_,p, *υ*, and g_i_). Due to the introduced k-epsilon turbulent closure, additional scaling conditions are required to be satisfied, as tabulated in Table 2. Depending on the underlying governing equations with specified initial and boundary conditions to hold in a scaled model, different self-similarity conditions could be achieved. For example, the conditions under which the Saint Venant equations system for unsteady open channel flow^31^, the conditions for the depth-averaged 2D hydrodynamic equations system^33^, and the conditions for the 3-dimensional incompressible Navier-Stokes equations for Newtonian fluids^34^ were reported recently.

Physical modeling is widely used in investigating fluid flows around hydraulic structures, airplanes, vehicles, machines, etc. The proposed Lie group scaling approach may improve the state of the art in physical modeling by providing a formal procedure for obtaining self-similarity in very complicated flow dynamics in time and space when the governing process, in terms of governing equations and initial and boundary conditions, is known.
